# Prevalence of and Risk Factors for Cognitive Impairment Among Elderly Without Cardio- and Cerebrovascular Diseases: A Population-Based Study in Rural China

**DOI:** 10.3389/fnagi.2018.00062

**Published:** 2018-03-28

**Authors:** Li Ren, Lingling Bai, Yanan Wu, Jingxian Ni, Min Shi, Hongyan Lu, Jun Tu, Xianjia Ning, Ping Lei, Jinghua Wang

**Affiliations:** ^1^Department of Neurology, Tianjin Medical University General Hospital, Tianjin, China; ^2^Tianjin Neurological Institute, Key Laboratory of Post-Neuroinjury Neuro-Repair and Regeneration in Central Nervous System, Ministry of Education and Tianjin City, Tianjin, China; ^3^Department of Epidemiology, Tianjin Neurological Institute, Tianjin, China; ^4^Department of Neurology, Liaocheng People’s Hospital, Liaocheng, China; ^5^Center of Clinical Epidemiology, Tianjin Medical University General Hospital, Tianjin, China; ^6^Department of Geriatrics, Tianjin Medical University General Hospital, Tianjin, China

**Keywords:** cognitive impairment, prevalence, risk factors, elderly, epidemiology

## Abstract

This study aimed to evaluate the prevalence of cognitive impairment and the distribution of its risk factors among residents aged ≥60 years without cardiovascular and cerebrovascular diseases in rural areas of northern China screened with the Chinese version of the Mini-Mental State Examination (MMSE). Between 2012 and 2013, a questionnaire survey was conducted to collect basic information from participants. Cognitive function was assessed using the MMSE. In the univariate analysis, risk factors for cognitive disorders were female sex, low education and central obesity, while drinking was found to be a protective factor. In the multivariate analysis, risk factors were old age (odds ratio [OR], 1.888; 95% confidence interval [CI]: 1.256–2.838; *P* = 0.002 for the 70-year-old group compared with the 60-year-old group; OR, 3.593; 95% CI, 2.468–5.230; *P* < 0.001 for the ≥75-year-old group compared with the 60-year-old group), low education (OR, 3.779; 95% CI: 2.218–6.440; *P* < 0.001 for the illiterate group compared with the group with ≥9 years of education; OR, 1.667; 95% CI, 1.001–2.775; *P* = 0.05 for the group with less than primary school compared with the group with ≥9 years of education), and higher blood pressure (BP; OR, 1.655; 95% CI: 1.076–2.544; *P* = 0.002 for individuals with stage III hypertension compared with those with normal BP). These findings suggest that it is crucial to manage and control level of BP, and improve educational attainment in order to reduce the prevalence and burden of cognitive impairment among low-income residents in rural China.

## Introduction

According to World Health Organization reports, there are approximately 47.5 million dementia patients worldwide, with 7.7 million newly diagnosed cases each year. It has been estimated that the annual global dementia-related healthcare costs were 604 billion USD (Wimo et al., 2013). The morbidity and prevalence of dementia, and the investment in dementia treatment and research, are unevenly distributed among developed and developing countries (Wimo et al., 2013). The proportion of dementia in developed countries is declining (Langa et al., 2017), accompanied by an increase in developing countries. In 2010, dementia in developing countries accounted for 58% of all cases worldwide, and it is predicted to reach 63% in 2030 and 71% in 2050 (Prince et al., 2013). Dementia has been ranked first among chronic non-communicable diseases in low-income and middle-income countries (Yang et al., 2008; Mayosi et al., 2009; Patel et al., 2011; Schmidt et al., 2011). The prevalence of cognitive impairment in rural areas is higher than that in urban areas (Tang et al., 2007; Gavrila et al., 2009; Nunes et al., 2010). There was a range of 18.9%–22.4% for cognitive impairment in people >60 years of age (Sherina et al., 2004; Laks et al., 2005; Lopes et al., 2007). In rural areas of northern China, the prevalence of dementia and Alzheimer’s disease in residents aged 60 years and older is 7.7% and 5.4%, respectively (Cristina et al., 2001), while that of cognitive impairment and dementia is 73.2% among the elderly aged 80 years and older (Ji et al., 2015). However, studies on the prevalence of and associated risk factors for non-vascular cognitive impairment are rare, especially in a population with low educational attainment. Thus, we aimed to explore the prevalence of and risk factors for non-vascular cognitive impairment based on the Mini-Mental State Examination (MMSE) in a low-income population in rural China.

## Materials and Methods

This was a population-based, cross-sectional study conducted from April 2014 to January 2015. The participants were from the Tianjin Brain Study, which has been described previously (Zhao et al., 2016; Ning et al., 2017). In brief, the total population included 14,251 individuals from 18 administrative villages. Approximately 95% of the population were low-income farmers, with a* per capita* disposable income of <1600 USD in 2014 (Zhao et al., 2016). In 2011, the average length of education was 5.26 years (Wang et al., 2015). All residents aged 60 years and older with normal vision and audition were recruited to this study. However, those with a registered previous history of myocardial infarction, stroke, depression, congenital hypophrenia and psychosis (including schizophrenia, paranoia schizophrenia) were excluded from the current study.

This study was carried out in accordance with the recommendations of the ethics committee of Tianjin Medical University General Hospital. All subjects gave written informed consent in accordance with the Declaration of Helsinki. The protocol was approved by the ethics committee of Tianjin Medical University General Hospital.

This study was conducted through face-to-face interviews by trained research staff. A pre-designed questionnaire was used to collect the following information: demographic information (including name, sex, date of birth and educational level), individual medical history (including the presence or occurrence of hypertension and diabetes mellitus), and lifestyle factors (including cigarette smoking and alcohol consumption).

A physical examination was performed to obtain information on height, weight, and waist circumference with thin clothes. Body mass index (BMI) was calculated as the individual’s weight (kg) divided by the square of the individual’s height (m^2^). Blood pressure (BP) measurements were performed using an electronic sphygmomanometer (HEM-741C, Omron, Tokyo, Japan). Subjects were asked to remain resting in a sitting position for 15 min before testing; BP was measured three times and the mean was obtained (Zhang et al., 2012). The levels of fasting blood glucose (FBG), total cholesterol (TC), triglycerides (TG), high-density lipoprotein cholesterol (HDL-C) and low-density lipoprotein cholesterol (LDL-C) were tested in the central laboratory of Tianjin Ji County People’s Hospital.

Carotid ultrasonography was also performed to evaluate carotid intima-media thickness (IMT). One trained technician blinded to participants’ information performed all ultrasound examinations. The patients were examined while they were in the supine position using B-mode ultrasonography (Terason 3000; Burlington, MA, USA) with a 5–12-MHz linear array transducer. The extracranial carotid artery trees (i.e., the common carotid artery, the bifurcation, and the internal and external carotid arteries) on both sides were screened for plaque. Images were obtained and digitally stored in accordance with a standard protocol. Both longitudinal and transverse dynamic images of each plaque were stored.

Owing to their high sensitivity and specificity of screening for dementia and cognitive impairment (Canadian Task Force on Preventive Health Care, 2016), we selected the MMSE and Montreal Cognitive Assessment (MoCA) scales. However, considering that the content of the MoCA was not suitable for individuals living in rural areas with low educational levels in China, we assessed cognitive impairment with the Chinese version of the MMSE. Furthermore, cognitive function was measured using the MMSE. All participants completed the MMSE scale under professional inquiry but by themselves.

We used the following diagnostic criteria for the variables of interest in our study: cognitive impairment was defined by an MMSE score of <17 in the illiterate group, <22 in the primary school group, and <26 in the junior school and above group. Hypertension was defined by an systolic blood pressure (SBP) ≥140 mmHg, diastolic blood pressure (DBP) ≥90 mmHg, the use of antihypertensive drugs, or a history of hypertension. Stage I hypertension was defined as 140 mmHg ≤ SBP < 160 mmHg, or 90 mmHg ≤ DBP < 100 mmHg; stage II hypertension was defined as 160 mmHg SBP < 180 mmHg, or 100 mmHg ≤ DBP < 110 mmHg; stage III hypertension was defined as SBP ≥180 mmHg or DBP ≥110 mmHg. Diabetes was defined as a FBG ≥ 7.0 mmol/L, taking medication for diabetes, or a self-reported history of diabetes. Obesity was defined as a BMI ≥28.0 kg/m^2^ and overweight was defined as 24.0 ≤ BMI < 28.0 kg/m^2^. Central obesity was defined as a waist circumference >102 cm for men and >88 cm for women. High TC was defined as ≥6.22 mmol/L; high TG was defined as ≥2.26 mmol/L; high LDL-C was defined as ≥4.14 mmol/L; and low HDL-C was defined as ≤1.04 mmol/L. Because carotid IMT was generally low in the present study, we classified it according to interquartile ranges; intimal thickening was defined as a thickness greater than the value of the 75% interquartile range. Plaques are focal structures that encroach into the arterial lumen by at least 0.5 mm or 50% of the surrounding IMT, or demonstrate a thickness of >1.5 mm, as measured from the intima-lumen interface to the media-adventitia interface. Smoking was defined as smoking ≥1 cigarette daily for more than 1 year. Drinking was defined as drinking >50 mL of alcohol at least once per week for more than 6 months.

The screening criteria for cognitive impairment were made based on an adjusted MMSE score. Cut-off points for cognitive impairment were set at 1 standard deviation below the mean of the MMSE according to educational level. The following were deemed positive for possible cognitive impairment: MMSE score <17 points in the illiterate group, <22 points in the primary school group, and <26 points in the secondary and above group (Nunes et al., 2010).

Continuous variables were presented as means and standard deviations, and the Student *t*-test was used to compare the differences between the two groups. Categorical variables were presented as numbers with frequencies, and the chi-squared test was performed to compare the differences between the two groups. All participants were categorized into four age groups (60–64, 65–69, 70–74 and ≥75 years), four education groups (0, 1–5, 6–8 and ≥9 years), and four blood pressure (BP) groups (normal, stage I hypertension, stage II hypertension, and stage III hypertension). The risk factors for cognitive impairment were assessed using logistic regression analyses, with cognitive impairment (yes or no) as the dependent variable and demographical features, previous histories of diseases, and risk factors as independent variables. Results of the univariate analysis were presented as unadjusted odds ratios (ORs) and 95% confidence intervals (CIs); results of the multivariate analysis were presented as adjusted ORs and 95% CIs after adjustment for covariates. *P* < 0.05 was considered statistically significant. SPSS for Windows (version 15.0; SPSS Inc., Chicago, IL, USA) was used for analyses.

## Results

**Figure 1:**
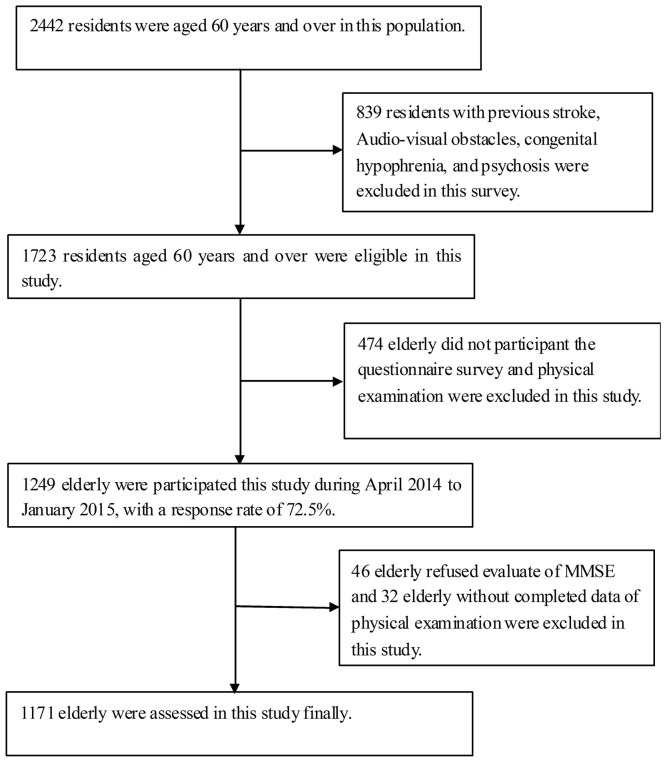
Flow chart of participants.

In this population, there were 2562 residents who were aged 60 years and older. Of these, 839 residents did not meet the inclusion criterion for this study. Thus, there were 1723 residents who were eligible to participate in this study. There were 1249 residents who participated in this study, for a response rate of 72.5%. Finally, 1171 elderly were assessed after excluding 46 residents who refused to participate in this investigation and 32 residents who did not complete the entire assessment (Figure 1).

Of the 1171 participants, men accounted for 45.7% and women accounted for 54.3%, with an average age of 66.84 years. The average educational achievement was 3.95 years overall, with an illiteracy rate of 27.8%. The general prevalence of risk factors was 79.4% for hypertension, 16.4% for diabetes, 20.3% for obesity, 34.4% for central obesity, 63.4% for smoking, and 30.6% for drinking, respectively. Moreover, there were 34.8% residents with stage I hypertension, 24.9% with stage II hypertension, and 15.0% with stage III hypertension (Table 1).

**Table 1:** The demographic characteristics of the study population.

Characteristics	Male	Female	Total
Numbers, *n* (%)	535 (45.7)	636 (54.3)	1171
Age, years, means (SD)	67.31 (6.69)	66.64 (6.83)	66.95 (6.78)
Age group, *n* (%):			
60 years	224 (41.9)	304 (47.8)	528 (45.1)
65 years	138 (25.8)	157 (24.7)	295 (25.2)
70 years	83 (15.5)	72 (11.3)	140 (13.2)
≥75 years	90 (16.8)	103 (16.2)	181 (16.5)
Education level, years (SD)	5.45 (2.74)	2.69 (3.00)	3.95 (3.19)
Education level, *n* (%):			
0 years	50 (9.3)	275 (43.2)	325 (27.8)
1 years	165 (30.8)	216 (34.0)	381 (32.5)
6 years	232 (43.4)	109 (17.1)	341 (29.1)
≥9 years	88 (16.4)	36 (5.7)	124 (10.6)
Current smoking, *n* (%)	391 (73.1)	351 (55.2)	742 (63.4)
Current drinking, *n* (%)	258 (48.2)	100 (15.7)	358 (30.6)
Hypertension, *n* (%)	423 (79.1)	507 (79.7)	930 (79.4)
Diabetes, *n* (%)	72 (13.5)	120 (18.9)	192 (16.4)
Central obesity, *n* (%)	169 (31.6)	128 (20.1)	297 (25.4)
Obesity, *n* (%)	323 (60.4)	323 (50.8)	646 (55.2)
Bp group, *n* (%):			
Normal BP	135 (25.2)	162 (25.5)	297 (25.4)
Stage I HBP	192 (35.9)	215 (33.8)	407 (34.8)
Stage II HBP	125 (23.4)	166 (26.1)	291 (24.9)
Stage III HBP	83 (15.5)	93 (14.6)	176 (15.0)
BMI, Kg/m^2^, means (SD)	24.68 (3.33)	25.32 (3.61)	25.03 (3.50)
WC, cm, means (SD)	88.81 (9.27)	89.39 (8.71)	89.13 (8.97)
SBP, mmHg, means (SD)	153.25 (23.01)	154.44 (23.03)	153.90 (23.02)
DBP, mmHg, means (SD)	87.26 (11.51)	85.60 (11.94)	86.36 (11.77)
FBG, mmol/L, means (SD)	5.84 (1.16)	6.16 (1.89)	6.01 (1.61)
TC, mmol/L, means (SD)	4.57 (0.96)	5.17 (1.06)	4.89 (1.06)
TG, mmol/L, means (SD)	1.43 (0.85)	1.83 (1.29)	1.65 (1.13)
HDL, mmol/L, means (SD)	1.42 (0.44)	1.52 (0.49)	1.47 (0.47)
LDL, mmol/L, means (SD)	2.50 (0.80)	2.84 (0.89)	2.68 (0.87)
IMT, cm, means (SD)	0.60 (0.10)	0.58 (0.08)	0.59 (0.09)

The raw prevalence of cognitive impairment was 32.4% overall, 25.6% in men and 38.1% in women. In the univariate analysis, the prevalence of cognitive impairment increased as age and BP increase but decreased as education level increased. Individuals with cognitive impairment were more likely to have hypertension and central obesity, but were less likely to smoke and drink alcohol (Table 2). Moreover, there was a higher age and SBP in the cognitive impairment group than in the normal group (Table 3).

**Table 2:** Distribution of risk factors by cognitive function.

Characteristics	Abnormal MMSE	*X*^2^	*P*
Gender, *n* (%):		20.551	<0.001
Male	137 (25.6)		
Female	242 (38.1)		
Age group, *n* (%):		72.287	<0.001
60 years	126 (23.9)		
65 years	83 (28.1)		
70 years	58 (37.4)		
≥75 years	112 (58.0)		
Education level, *n* (%):		96.213	<0.001
0 year	175 (53.8)		
1 years	121 (31.8)		
6 years	58 (17.0)		
≥9 years	25 (20.2)		
Hypertension, *n* (%):		4.679	0.031
No	64 (26.6)		
Yes	315 (33.9)		
Diabetes, *n* (%):		0.672	0.412
No	312 (31.9)		
Yes	67 (34.9)		
Current smoking, *n* (%):		3.858	0.058
No	154 (35.9)		
Yes	225 (30.3)		
Current drinking, *n* (%):		6.543	0.011
No	282 (34.7)		
Yes	97 (27.1)		
Central obesity, *n* (%):		5.958	0.015
No	230 (29.9)		
Yes	149 (37.0)		
Obesity, *n* (%):		0.221	0.638
No	305 (32.7)		
Yes	74 (31.1)		
IMT thickening, *n* (%):		0.201	0.654
No	286 (32.7)		
Yes	93 (31.3)		
Plaque presence, *n* (%):		3.056	0.080
No	156 (29.7)		
Yes	223 (34.5)		
Bp group, *n* (%):		17.230	<0.001
Normal BP	82 (27.6)		
Stage I HBP	114 (28.0)		
Stage II HBP	105 (36.1)		
Stage III HBP	78 (44.3)		

**Table 3:** Differences in physical examination items by cognitive function groups.

Items	Normal MMSE	Abnormal MMSE	*t*	*P*
SBP, mmHg (SD)	152.05 (21.98)	157.77 (24.64)	4.001	<0.001
DBP, mmHg (SD)	86.33 (11.22)	86.42 (12.87)	0.120	0.905
FBG, mmol/L (SD)	5.99 (1.63)	6.06 (1.55)	0.648	0.517
TC, mmol/L (SD)	4.86 (1.06)	4.96 (1.06)	1.591	0.112
TG, mmol/L (SD)	1.65 (1.18)	1.65 (1.00)	0.083	0.934
HDL, mmol/L (SD)	1.46 (0.46)	1.51 (0.50)	1.600	0.110
LDL, mmol/L (SD)	2.67 (0.87)	2.71 (0.86)	0.585	0.558
Average IMT, mm (SD)	0.59 (0.09)	0.59 (0.08)	0.218	0.827

Table 4 shows that age, education level, and higher BP were associated with cognitive impairment. The corresponding ORs (95% CIs) were 1.89 (1.26–2.84; *P* = 0.002) for 70-year-old individuals and 3.59 (2.47–5.23; *P* < 0.001) for individuals ≥75 years old compared with 60-year-old individuals; 3.78 (2.22–6.44; *P* < 0.001) for the illiterate group and 1.67 (1.00–2.78; *P* = 0.050) for those with 1–5 years education level compared with those with ≥9 years of education; and 1.66 (1.08–2.54; *P* = 0.002) for those with stage III hypertension compared to those with normal BP.

**Table 4:** Determinants of cognitive impairment using multivariate logistic regression analysis.

Characteristics	Reference	OR (95%CI)	*P*
Gender:	Female		
Male		1.03 (0.72, 1.48)	0.855
Age group:	60–64 years		
65–69 years		1.36 (0.97, 1.91)	0.079
70–74 years		1.89 (1.26, 2.84)	0.002
≥75 years		3.59 (2.47, 5.23)	<0.001
Education level:	≥9 years		
0 year		3.78 (2.22, 6.44)	<0.001
1–5 years		1.67 (1.00, 2.78)	0.050
6–8 years		0.79 (0.46, 1.35)	0.384
Current drinking	Never		
Yes		0.89 (0.65, 1.22)	0.474
Central obesity	No		
Yes		1.14 (0.83, 1.58)	0.422
BP group:	Normal BP		
Stage I HBP		0.97 (0.67, 1.39)	0.853
Stage II HBP		1.45 (0.99, 2.12)	0.056
Stage III HBP		1.66 (1.08, 2.54)	0.022

## Discussion

This is the first study to examine the prevalence of and relevant risk factors for cognitive impairment among a low-income elderly population without cardiovascular and cerebrovascular diseases in rural China. The prevalence of cognitive impairment was 32.37% among individuals aged 60 years and older, 25.6% in men and 38.1% in women. Age, education level and BP levels were significantly associated with cognitive impairment. Compared to individuals aged 60 years and older, the prevalence of non-vascular cognitive impairment increased remarkably among those aged 70 years and older; a similar trend was found in those with education levels of <6 years, compared to individuals with education levels of 9 years or more. Moreover, there was a greater prevalence of cognitive impairment among patients with stage III hypertension than among those with normal BP.

The prevalence of dementia and cognitive impairment has been inconsistent in different regions and subjects, with a range of 13.1% to 25.7% (Cristina et al., 2001; Sherina et al., 2004; Lopes et al., 2007; Shi et al., 2013; Ji et al., 2015; Langa et al., 2017). However, few studies have reported the prevalence of cognitive impairment among a low-income elderly population without cardiovascular and cerebrovascular diseases. In this study, the prevalence of cognitive impairment was surprisingly high, up to 32.4%. The low level of education and income in this population may partially explain this finding.

The sex disparity in cognition impairment has been established. Population studies from Malaysia reported that women had worse cognitive function than men did (Sherina et al., 2004). Neuropsychological screening results from northern Italy showed that the prevalence of cognitive impairment was higher in women, even after accounting for age (Cristina et al., 2001). Population studies from rural northern China indicated that women were more likely to experience cognitive impairment than men were (Shi et al., 2013; Ji et al., 2015). In previous studies, the prevalence of cognitive impairment was 2.3-fold higher in women than in men among people aged 60 years old and older (Ji et al., 2015) and 1.8-fold higher in women than in men among people aged 80 years old and older (Shi et al., 2013). The loss of estrogen in menopause may contribute the high prevalence of cognitive impairment in women (Sherina et al., 2004). However, several studies have shown that cognition was not related to sex (Lobo et al., 2005; Zhang et al., 2012; Bai et al., 2016). In the present study, cognition was worse in women than in men in the univariate analysis, but the sex difference disappeared in the multivariate analysis. Low educational attainment may account for the poor cognitive function in women in this population.

Age and education level were risk factors for cognitive impairment (Friedland, 1993; Elias et al., 1997; Ganguli et al., 2000; Lobo et al., 2005; Ngandu et al., 2007; Zhang et al., 2012; Shi et al., 2013; Bai et al., 2016). Consistent with previous studies, the prevalence of cognitive impairment in this elderly population increased remarkably among those aged 70 years and older compared to that in individuals aged 60–70 years; a similar trend was found in those with education levels of <6 years compared to that in individuals with education levels of 9 years or more, with a 3.8-fold increase in the illiterate group and a 1.7-fold increase in those with 1–5 years of education.

Most studies have shown a negative correlation between BP and cognition (Kivipelto et al., 2001; Whitmer et al., 2005; Peters et al., 2008; Gao et al., 2009; Oveisgharan and Hachinski, 2010; Higuchi et al., 2015), although some studies have shown that they were not related (Rönnemaa et al., 2011; Yang et al., 2011). Midlife arterial hypertension (SBP ≥160 mmHg) significantly increased the late-life risk of Alzheimer’s disease (Kivipelto et al., 2001; Whitmer et al., 2005; Higuchi et al., 2015). Hypertension has also been shown to predict progression to dementia in older people (Oveisgharan and Hachinski, 2010). The risk of dementia in the untreated hypertension group was higher than that in the treated group (Peters et al., 2008; Gao et al., 2009). In the present study, we divided BP into four grades: normal BP, stage I hypertension, stage II hypertension, and stage III hypertension. Stage III hypertension had a significant negative association with cognition. Because of the lower frequency of medication use in this study (Wang et al., 2014), there was a stronger negative correlation between hypertension and MMSE score. However, the exact mechanism for this relationship is not clear and needs to be studied further.

Associations between conventional risk factors and cognition has not been conclusive (Knopman et al., 2001; Musen et al., 2008; Solomon et al., 2009). There were no significant associations between FBG, blood lipids, and cognition in this study. Several studies have demonstrated that carotid intima-medial thickness (IMT) and carotid plaques were negatively correlated with cognitive function (van Oijen et al., 2007; Wendell et al., 2009; Sander et al., 2010; Zhong et al., 2011, 2012). Inconsistent with these findings, there was no relationship between carotid atherosclerosis and cognitive function in this study. A thin carotid IMT (0.60 mm for men and 0.58 mm for women) in our study compared with that in another study from China and the high frequency of carotid plaques (47.1% for men and 35.4% for women) may explain this disparity (Su et al., 2012; Zhan et al., 2016).

The following are limitations of this study. First, the study comprised a low-income, low-education rural population in northern China. Thus, its representation and generalizability are poor. Second, the present study only screened for cognitive impairment with MMSE scores and did not use other instruments to replace the MMSE for the evaluation of cognitive function; therefore, further exploratory research of the validity of this instrument of cognitive function is needed. However, this study was not designed to investigate the prevalence of and risk factors for dementia and different dementia types. Third, subjects with a history of depression and mental illness were excluded, rather than being screened with the Hamilton Depression Scale. Lastly, we only evaluated the above risk factors for cognition and did not consider the effects of medication history, family history, exercise, or social interaction on cognitive function. Finally, this was a cross-section study and so causation could not be identified.

In conclusion, the prevalence of cognitive impairment decreased with increasing education level, while it increased in older people and in those with hypertension. Subjects with stage III hypertension had a higher risk of cognitive impairment than did those with normal BP. The lower average educational level generally and the lower frequency of using antihypertensive drugs in this study population may have contributed to these results. These findings suggest that it is crucial to manage and control level of BP, and improve educational attainment among low-income populations in order to reduce the prevalence and burden of cognitive impairment in China.

## Author Contributions

JW, XN and PL contributed to the conception and design of the work and contributed in revising the work for important intellectual content. LR, LB, YW, JN, MS, HL and JT contributed to the data acquisition. JW and XN contributed to the analysis and interpretation of data for the work. LR and LB contributed in drafting the work. All authors approved of the final version to be published, and agree to be accountable for all aspects of the work in ensuring that questions related to the accuracy or integrity of any part of the work are appropriately investigated and resolved.

## Conflict of Interest Statement

The authors declare that the research was conducted in the absence of any commercial or financial relationships that could be construed as a potential conflict of interest.

## Abbreviations

BMI: body mass index
BP: blood pressure
CI: confidence intervals
CIND: cognitive impairment not dementia
CVD: cardiovascular disease
DBP: diastolic blood pressure
FBG: fasting blood glucose
HDL-C: high-density lipoprotein cholesterol
IMT: intima-media thickness
LDL-C: low-density lipoprotein cholesterol
MMSE: Mini-Mental State Examination
OR: odd ratios
SBP: systolic blood pressure
TC: total cholesterol
TG: triglycerides.

## References

[B1] Bai J. Wei P. Zhao N. Xiao Y. Yang C. Zhong J.. (2016). A study of mild cognitive impairment in veterans: role of hypertension and other confounding factors. Neuropsychol. Dev. Cogn. B Aging Neuropsychol. Cogn. 23, 703–715. 10.1080/13825585.2016.1161000 26999624

[B2] Canadian Task Force on Preventive Health Care Pottie K. Rahal R. Jaramillo A. Birtwhistle R. Thombs B. D.. (2016). Recommendations on screening for cognitive impairment in older adults. CMAJ 188, 37–46. 10.1503/cmaj.141165 26622001 PMC4695353

[B3] Cristina S. Nicolosi A. Hauser W. A. Leite M. L. Gerosa E. Nappi G. (2001). The prevalence of dementia and cognitive deficit in a rural population of 2442 residents in Northern Italy. A door-to-door survey. Eur. J. Neurol. 8, 595–600. 10.1046/j.1468-1331.2001.00301.x 11784344

[B4] Elias M. F. Elias P. K. D’Agostino R. B. Silbershatz H. Wolf P. A. (1997). Role of age, education, and gender on cognitive performance in the Framingham Heart Study:community-based norms. Exp. Aging Res. 23, 201–235. 10.1080/03610739708254281 9248817

[B5] Friedland R. P. (1993). Epidemiology, education, and the ecology of Alzheimer’s disease. Neurology 43, 246–249. 10.1212/WNL.43.2.246 8437687

[B6] Ganguli M. Dodge H. H. Chen P. Belle S. DeKosky S. T. (2000). Ten-year incidence of dementia in a rural elderly US community population :the MoUIES Project. Neurology 4, 1109–1116. 10.1212/WNL.54.5.1109 10720283

[B7] Gao S. Jin Y. Unverzagt F. W. Liang C. Hall K. S. Ma F.. (2009). Hypertension and cognitive decline in rural elderly chinese. J. Am. Geriatr. Soc. 57, 1051–1057. 10.1111/j.1532-5415.2009.02267.x 19507297 PMC2849159

[B8] Gavrila D. Antúnez C. Tormo M. J. Carles R. García Santos J. M. Parrilla G.. (2009). Prevalence of dementia and cognitive impairment in Southeastern Spain: the Ariadna study. Acta Neurol. Scand. 120, 300–307. 10.1111/j.1600-0404.2009.01283.x 19832772

[B9] Higuchi M. Chen R. Abbott R. D. Bell C. Launer L. Ross G. W.. (2015). Mid-life proteinuria and late-life cognitive function and dementia in elderly men: the Honolulu-Asia Aging Study. Alzheimer Dis. Assoc. Disord. 29, 200–205. 10.1097/WAD.0000000000000082 25626635 PMC4514569

[B10] Ji Y. Shi Z. Zhang Y. Liu S. Liu S. Yue W.. (2015). Prevalence of dementia and main subtypes in rural northern China. Dement. Geriatr. Cogn. Disord. 39, 294–302. 10.1159/000375366 25792116 PMC4993106

[B11] Kivipelto M. Helkala E. L. Laakso M. P. Hänninen T. Hallikainen M. Alhainen K.. (2001). Midlife vascular risk factors and Alzheimer’s disease in later life:longitudinal, population based study. BMJ 322, 1447–1451. 10.1136/bmj.322.7300.1447 11408299 PMC32306

[B12] Knopman D. Boland L. L. Mosley T. Howard G. Liao D. Szklo M.. (2001). Cardiovascular risk factors and cognitive decline in middle-aged adults. Neurology 56, 42–48. 10.1212/WNL.56.1.42 11148234

[B13] Laks J. Batista E. M. Guilherme E. R. Contino A. L. Faria M. E. Rodrigues C. S.. (2005). Prevalence of cognitive and functional impairment in community-dwelling elderly: importance of evaluating activities of daily living. Arq. Neuropsiquiatr. 63, 207–212. 10.1590/s0004-282x2005000200003 16100963

[B14] Langa K. M. Larson E. B. Crimmins E. M. Faul J. D. Levine D. A. Kabeto M. U.. (2017). A comparison of the prevalence of dementia in the united states in 2000 and 2012. JAMA Intern. Med. 177, 51–58. 10.1001/jamainternmed.2016.6807 27893041 PMC5195883

[B15] Lobo A. Saz P. Marcos G. Día J.-L. De-la-Cámara C. Ventura T. (2005). The *ZARADEMP* Project on the incidence, prevalence and risk factors of dementia (and depression) in the elderly community: II. Methods and first results. Eur. J. Psychiatry 19, 40–54. 10.4321/s0213-61632005000100004

[B16] Lopes M. A. Hototian S. R. Bustamante S. E. Azevedo D. Tatsch M. Bazzarella M. C.. (2007). Prevalence of cognitive and functional impairment in a community sample in Ribeirão Preto, Brazil. Int. J. Geriatr. Psychiatry 22, 770–776. 10.1002/gps.1737 17173353

[B17] Mayosi B. M. Flisher A. J. Lalloo U. G. Sitas F. Tollman S. M. Bradshaw D. (2009). The burden of non-communicable diseases in South Africa. Lancet 374, 934–947. 10.1016/S0140-6736(09)61087-4 19709736

[B18] Musen G. Jacobson A. M. Ryan C. M. Cleary P. A. Waberski B. H. Weinger K.. (2008). Impact of diabetes and its treatment on cognitive function among adolescents who participated in the diabetes control and complications trial. Diabetes Care 31, 1933–1938. 10.2337/dc08-0607 18606979 PMC2551630

[B19] Ngandu T. von Strauss E. Helkala E. L. Winblad B. Nissinen A. Tuomilehto J.. (2007). Education and dementia: what lies behind the association? Neurology 69, 1442–1450. 10.1212/01.WNL.0000277456.29440.16 17909157

[B20] Ning X. Sun J. Jiang R. Lu H. Bai L. Shi M.. (2017). Increased stroke burdens among the low- income young and middle aged in rural china. Stroke 48, 77–83. 10.1161/STROKEAHA.116.014897 27924051

[B21] Nunes B. Silva R. D. Cruz V. T. Roriz J. M. Pais J. Silva M. C. (2010). Prevalence and pattern of cognitive impairment in rural and urban populations from Northern Portugal. BMC Neurol. 10:42. 10.1186/1471-2377-10-42 20540726 PMC2905352

[B22] Oveisgharan S. Hachinski V. (2010). Hypertension, executive dysfunction, and progression to dementia: the canadian study of health and aging. Arch. Neurol. 67, 187–192. 10.1001/archneurol.2009.312 20142526

[B23] Patel V. Chatterji S. Chisholm D. Ebrahim S. Gopalakrishna G. Mathers C.. (2011). Chronic diseases and injuries in India. Lancet 377, 413–428. 10.1016/S0140-6736(10)61188-9 21227486

[B24] Peters R. Beckett N. Forette F. Tuomilehto J. Clarke R. Ritchie C.. (2008). Incident dementia and blood pressure lowering in the hypertension in the very elderly trial cognitive function assessment (HYVET-COG): a double-blind, placebo controlled trial. Lancet Neurol. 7, 683–689. 10.1016/S1474-4422(08)70143-1 18614402

[B25] Prince M. Bryce R. Albanese E. Wimo A. Ribeiro W. Ferri C. P. (2013). The global prevalence of dementia: a systematic review and metaanalysis. Alzheimers Dement. 9, 63–75. 10.1016/j.jalz.2012.11.007 23305823

[B26] Rönnemaa E. Zethelius B. Lannfelt L. Kilander L. (2011). Vascular risk factors and dementia: 40-year follow-up of a population based cohort. Dement. Geriatr. Cogn. Disord. 31, 460–466. 10.1159/000330020 21791923

[B27] Sander K. Bickel H. Förstl H. Etgen T. Briesenick C. Poppert H.. (2010). Carotid- intima media thickness is independently associated with cognitive decline. Int. J. Geriatr. Psychiatry 25, 389–394. 10.1002/gps.2351 19750556

[B28] Schmidt M. I. Duncan B. B. Azevedo e Silva G. Menezes A. M. Monteiro C. A. Barreto S. M.. (2011). Chronic non-communicable diseases in Brazil: burden and current challenges. Lancet 377, 1949–1961. 10.1016/S0140-6736(11)60135-9 21561658

[B29] Sherina M. S. Rampal L. Mustaqim A. (2004). Cognitive impairment among the elderly in a rural community in malaysia. Med. J. Malaysia 59, 252–257. 15559177

[B30] Shi Z. Zhang Y. Yue W. Liu M. Huo Y. R. Liu S.. (2013). Prevalence and clinical predictors of cognitive impairment in individuals aged 80 years and older in rural China. Dement. Geriatr. Cogn. Disord. 36, 171–178. 10.1159/000350811 23900137

[B31] Solomon A. Kivipelto M. Wolozin B. Zhou J. Whitmer R. A. (2009). Midlife serum cholesterol and increased risk of Alzheimer’s and vascular dementia three decades later. Dement. Geriatr. Cogn. Disord. 28, 75–80. 10.1159/000231980 19648749 PMC2814023

[B32] Su T. C. Chien K. L. Jeng J. S. Chen M. F. Hsu H. C. Torng P. L.. (2012). Age- and gender-associated determinants of carotid intima-media thickness a community-based study. J. Atheroscler. Thromb. 19, 872–880. 10.5551/jat.10728 22972311

[B33] Tang Z. Zhang X. Wu X. Liu H. Diao L. Guan S. (2007). Prevalence of the mild cognitive impairment among elderly in Beijing. Chin. Ment. Health J. 21, 116–118.

[B34] van Oijen M. de Jong F. J. Witteman J. C. Hofman A. Koudstaal P. J. Breteler M. M. (2007). Atherosclerosis and risk for dementia. Ann. Neurol. 61, 403–410. 10.1002/ana.21073 17328068

[B35] Wang J. An Z. Li B. Yang L. Tu J. Gu H.. (2015). Increasing stroke incidence and prevalence of risk factors in a low-income Chinese population. Neurology 84, 374–381. 10.1212/WNL.0000000000001175 25540314

[B36] Wang J. Ning X. Yang L. Lu H. Tu J. Jin W.. (2014). Trends of hypertension prevalence, awareness, treatment and control in rural areas of northern China during 1991–2011. J. Hum. Hypertens. 28, 25–31. 10.1038/jhh.2013.44 23739160

[B37] Wendell C. R. Zonderman A. B. Metter E. J. Najjar S. S. Waldstein S. R. (2009). Carotid intimal medial thickness predicts cognitive decline among adults without clinical vascular disease. Stroke 40, 3180–3185. 10.1161/STROKEAHA.109.557280 19644063 PMC2753681

[B38] Whitmer R. A. Sidney S. Selby J. Johnston S. C. Yaffe K. (2005). Midlife cardiovascular risk factors and risk of dementia in late life. Neurology 64, 277–281. 10.1212/01.WNL.0000149519.47454.f2 15668425

[B39] Wimo A. Jönsson L. Bond J. Prince M. Winblad B. Alzheimer Disease International. (2013). The worldwide economic impact of dementia 2010. Alzheimers Dement. 9, 1.e3–11.e3. 10.1016/j.jalz.2012.11.006 23305821

[B40] Yang G. Kong L. Zhao W. Wan X. Zhai Y. Chen L. C.. (2008). Emergence of chronic non-communicable disease in China. Lancet 372, 1697–1705. 10.1016/S0140-6736(08)61366-5 18930526

[B41] Yang Y. H. Roe C. M. Morris J. C. (2011). Relationship between late-life hypertension, blood pressure and Alzheimer’s disease. Am. J. Alzheimers Dis. Other Demen. 26, 457–462. 10.1177/1533317511421779 21921085 PMC3312309

[B42] Zhan C. Shi M. Yang Y. Pang H. Fei S. Bai L.. (2016). Prevalence and risk factors of carotid plaque among middle-aged and elderly adults in rural tianjin, china. Sci. Rep. 6:23870. 10.1038/srep23870 27029785 PMC4814923

[B43] Zhang Y. Xu Y. Nie H. Lei T. Wu Y. Zhang L.. (2012). Prevalence of dementia and major dementia subtypes in the Chinese populations: a meta-analysis of dementia prevalence surveys, 1980–2010. J. Clin. Neurosi. 19, 1333–1337. 10.1016/j.jocn.2012.01.029 22682650

[B44] Zhao W. Wu Y. Shi M. Bai L. Tu J. Guo Z.. (2016). Sex differences in prevalence of and risk factors for carotid plaque among adults: a population-based cross-sectional study in rural china. Sci. Rep. 6:38618. 10.1038/srep38618 27922121 PMC5138635

[B45] Zhong W. Cruickshanks K. J. Huang G. H. Klein B. E. Klein R. Nieto F. J.. (2011). Carotid atherosclerosis and cognitive function in midlife: the beaver dam offspring study. Atherosclerosis 219, 330–333. 10.1016/j.atherosclerosis.2011.07.013 21831374 PMC3206159

[B46] Zhong W. Cruickshanks K. J. Schubert C. R. Acher C. W. Carlsson C. M. Klein B. E.. (2012). Carotid atherosclerosis and 10-year changes in cognitive function. Atherosclerosis 224, 506–510. 10.1016/j.atherosclerosis.2012.07.024 22854188 PMC3459157

